# Stevioside Improves Antioxidant Capacity and Intestinal Barrier Function while Attenuating Inflammation and Apoptosis by Regulating the NF-κB/MAPK Pathways in Diquat-Induced Oxidative Stress of IPEC-J2 Cells

**DOI:** 10.3390/antiox12051070

**Published:** 2023-05-10

**Authors:** Qinglei Xu, Mingzheng Liu, Xiaohuan Chao, Chunlei Zhang, Huan Yang, Jiahao Chen, Bo Zhou

**Affiliations:** College of Animal Science and Technology, Nanjing Agricultural University, Nanjing 210095, China; 2019205004@njau.edu.cn (Q.X.); 2020205018@stu.njau.edu.cn (M.L.); 2021205020@stu.njau.edu.cn (X.C.); 2020105039@stu.njau.edu.cn (C.Z.); 2021105038@stu.njau.edu.cn (H.Y.); 2021105039@stu.njau.edu.cn (J.C.)

**Keywords:** stevioside, antioxidant, oxidative stress, inflammation, IPEC-J2 cells, NF-κB/MAPK signaling pathway

## Abstract

As a natural sweetener, stevioside is extracted from *Stevia rebaudiana* Bertoni and possesses potent antioxidant activity. However, little information is known about its protective role in maintaining the intestinal epithelial cells health under oxidative stress. The aim of this study was to investigate the protective effects and underlying mechanisms of stevioside on alleviating inflammation, apoptosis, and improving antioxidant capacity in intestinal porcine epithelial cells (IPEC-J2) under oxidative stress by diquat. The results demonstrated that the pretreatment with stevioside (250 μM) for 6 h increased cell viability and proliferation and prevented apoptosis induced by diquat at 1000 μM for 6 h in IPEC-J2 cells, compared with the diquat alone-treated cells. Importantly, stevioside pretreatment significantly reduced ROS and MDA production as well as upregulated T-SOD, CAT, and GSH-Px activity. Moreover, it also decreased cell permeability and improved intestinal barrier functions by significantly upregulating the tight junction protein abundances of claudin-1, occludin, and ZO-1. At the same time, stevioside significantly down-regulated the secretion and gene expression of IL-6, IL-8, and TNF-α and decreased the phosphorylation levels of NF-κB, IκB, and ERK1/2 compared with the diquat alone group. Taken together, this study demonstrated that stevioside alleviated diquat-stimulated cytotoxicity, inflammation, and apoptosis in IPEC-J2 cells, protecting cellular barrier integrity and mitigating oxidative stress by interfering with the NF-κB and MAPK signaling pathways.

## 1. Introduction

Oxidative stress (OS) arises as a consequence of an imbalance between oxidation and antioxidant levels in the body [1]. Oxidative stress refers to the excessive production of reactive oxygen species (ROS) and reactive nitrogen species (RNS) relative to antioxidant defense, resulting in the disorder of free radical metabolism in the body [2]. The physiological function of piglets is not fully developed at weaning, and their ability to provide antioxidant defense is weak [3]. The intestinal tract of pigs is composed of monolayer intestinal epithelial cells, which play an important role in the digestion and absorption of nutrients, immune barrier function, and amino acid metabolism [4]. When piglets are subjected to stress, the degree of the oxidation exceeds the clearance rate of oxides, and the imbalance of oxidation system and antioxidant system causes oxidative stress and then leads to intestinal epithelial damage [5]. Oxidative stress causes the intestinal barrier function of piglets to be damaged, which affects the digestion and absorption of nutrients, and the immune function is decreased, which leads to the decline of disease resistance and even diarrhea [6]. In addition, oxidative stress is common in the intensive production of pigs, which poses greater risks to animal health and seriously affects the economic benefits of the pig industry. Therefore, it is very important to develop effective methods to protect pigs against oxidative injury caused by oxidative stress.

Stevioside is a natural sweetener extracted from *Stevia rebaudiana* Bertoni that is often used as a sweetener additive [7]. Natural noncaloric sweeteners that can substitute for sucrose in the food industry have attracted more and more attention with the rising incidence of obesity and diabetes [8]. In addition to the application of stevioside as a sweetener in food, stevioside also has the pharmacological effects of hypoglycemic [9], antioxidant [10], anti-inflammatory activities [11] and immune regulation [12]. In addition, the abuse of antibiotics causes antibiotic residues in foodstuffs, which led to the ban of antibiotics as feed additives in many countries [13]. Stevioside, as a natural feed additive, has attracted widespread attention from researchers. Previous studies have shown that stevioside as a feed additive has positive potential to improve goat feed intake [14]. Furthermore, it should be noted that the administration of stevioside has been shown to yield favorable outcomes in terms of animal health and growth. This is exemplified by the fact that the provision of stevioside to maternal subjects resulted in an increase in innate immunity and a notable improvement in the physiological state of subsequent generations of chicken offspring [15]. Stevioside alleviated the intestinal mucosal damage of broilers caused by lipopolysaccharide (LPS) through its anti-inflammatory and antioxidant activities [16]. At the same time, supplementary stevioside in cattle diets improved growth performance, carcass traits, meat quality, and decreased the lipid oxidation of beef during storage [17]. The incorporation of stevioside into the diet of broiler chickens resulted in improved growth performance. As such, stevioside may be considered a viable option for use as a growth-promoting feed additive in broiler chickens [18]. Our previous study showed that 0.5% stevioside in acidified water improved the GSH activity of weaned piglets and showed an antioxidant effect [19]. However, the underlying molecular mechanism of how stevioside exerts an antioxidant effect in pigs remains unknown.

Diquat, as a classical inducer of oxidative stress, is widely used to establish an oxidative stress model in animals [20]. Intestinal epithelial cells participate in digestion, absorption, secretion, barrier integrity, and the stress response of the intestine [21]. The IPEC-J2 cell line was derived from the jejunum epithelium of newborn piglets and is often be used as an in vitro model of small intestinal epithelial cells in pigs [22]. Despite the potential benefits attributed to stevioside as an antioxidant, its molecular mechanism of action and the extent to which it may be applied in the context of oxidative stress remain ambiguous, particularly within the confines of IPEC-J2 cell lines. Thus, the purposes of this study were to investigate the antioxidant effects of stevioside against oxidative damage induced by diquat in IPEC-J2 cells and to explore the underlying molecular mechanism.

## 2. Materials and Methods

### 2.1. Chemicals

Diquat (purity ≥ 99.9%) was purchased from TMRM (Tan-Mo Technology Co., Ltd., Changzhou, China). Stevioside standards (purity ≥ 98% by high performance liquid chromatography, CAS: 57817-89-7) were purchased from Shanghai Yuanye Bio-Technology Co., Ltd. (Shanghai, China). Dulbecco’s modified Eagle’s medium/nutrient mixture F12 (DMEM/F12) medium, fetal bovine serum (FBS), trypsin, and penicillin-streptomycin were purchased from Gibco (Grand Island, NY, USA). The antibodies used here were obtained from ABclonal Technology (ABclonal, Wuhan, China).

### 2.2. Cell Culture and Treatments

The IPEC-J2 cells were kindly provided by Prof. Chunmei Li (Nanjing Agricultural University, Nanjing, China), cultured in DMEM/F12 medium containing 10% fetal bovine serum (FBS) and 1% penicillin-streptomycin, and maintained at 37 °C with a 5% CO_2_ atmosphere. Cells were processed as follows: control group (CON), stevioside group (ST), diquat group (DQ), and stevioside + diquat group (ST + DQ). Specifically, the control cells were cultivated in normal DMEM/F12 containing 10% FBS (CON). Cells were exposed to optimum concentration diquat alone for optimum treatment durations in the diquat treatment group (DQ), while those in the stevioside treatment group (ST) were exposed to optimum concentration stevioside for optimum treatment durations. Cells were pretreated with optimum concentration stevioside for optimum treatment durations, followed by co-incubation with optimum concentration diquat for optimum treatment durations in the stevioside + diquat treatment group (ST + DQ).

### 2.3. Cell Viability Assay

IPEC-J2 cells were cultured in 96-well plates (Costar, Corning Inc., Corning, NY, USA) for diquat or stevioside treatment. Firstly, IPEC-J2 cells were treated with diquat to establish an oxidative stress model in vitro. The cytotoxicity of diquat was evaluated using the methyl thiazolyl tetrazolium (MTT) assay (Nanjing Jiancheng Bioengineering Institute, Nanjing, China). IPEC-J2 cells were seeded in 96-well culture plates at 1 × 10^4^ cells/well, and 100 µL of complete culture medium was added to culture overnight. After 4, 6, or 8 h of treatment with diquat at final concentrations of 0, 100, 250, 500, 750, 1000, 1250, and 1500 μM, incubated cells with 1× MTT working solution for 4 h at 37 °C. The absorbance at 570 nm was measured using a microplate reader (Tecan, Austria GmbH, Grödig, Austria). Using the 50% inhibitory concentration (IC 50) as a standard, we selected the optimal concentration of diquat to simulate oxidative stress in IPEC-J2 cells.

After growing to 80% confluence in 96-well plates, the cells were pretreated with 0, 50, 100, 250, and 500 µM stevioside for 4, 6, or 8 h. After incubation with optimum stevioside for optimum treatment durations, cells were treated with stevioside + diquat for optimum treatment durations. To analyze cell viability, cells were incubated with CCK-8 solution (Vazyme, Nanjing, China), and the absorbance at 450 nm was measured using a microplate reader (Tecan, Austria GmbH, Grödig, Austria). Cell viability was shown as a percentage of viable cells compared to the wells containing control cells not exposed to stevioside or diquat.

### 2.4. Measurement of Cell Proliferation

To assess cell proliferation, the Cell-light™ EdU Apollo^®^ 567 In Vitro Imaging Kit (manufactured by Ribobio, Guangzhou, China) was employed and implemented in accordance with the recommended guidelines. Cellular staining was observed with fluorescence microscopy (Zeiss, LSM 700; Oberkochen, Germany). Proliferation was quantified and shown as the percentage of EdU-positive cells.

### 2.5. Flow Cytometric Determination of Cell Apoptosis

Cells were treated as described above (CON, ST, DQ, and ST + DQ). Apoptosis analysis was performed using an Annexin V-FITC/PI Apoptosis Detection Kit (Vazyme, Nanjing, China). In brief, the cells were collected from a 12-well culture plate, and then they were incubated with 5 μL annexin V-FITC and 5 μL propidium iodide at room temperature for 20 min in the dark. Then, the stained cells were analyzed by a flow cytometer (BD Biosciences, San Jose, CA, USA). Apoptotic cells were expressed as a percentage of total cells.

### 2.6. Measurement of Intracellular ROS Production

IPEC-J2 cells (10^4^ cells per well) were seeded in 96-well plates with 8 parallel holes in each group. Intracellular reactive oxygen species (ROS) production was monitored using a ROS assay kit (Nanjing Jiancheng Bioengineering Institute, Nanjing, China). In brief, IPEC-J2 cells were stained with 10 µM 2′,7′-dichlorohydro-fluorescein diacetate (DCFH-DA) for 30 min at 37 °C, then washed with FBS-free media and re-suspended in PBS. Intracellular ROS production was determined with excitation at 480 nm and emission at 530 nm using a microplate reader (Tecan, Austria GmbH, Grödig, Austria). Its fluorescence signal intensity is proportional to the ROS levels in IPEC-J2 cells. The intracellular ROS levels were expressed as relative fluorescence signals and then normalized to the control group.

### 2.7. Determination of Oxidative Stress Parameters

After the IPEC-J2 cells were treated as described above, the cells were carefully washed twice with PBS and lysed with RIPA Lysis Buffer (containing PMSF) (Solarbio, Beijing, China) for 10 min. Centrifuge at 1000× *g* for 10 min at 4 °C and collect the supernatants. Then, the activities of total superoxide dismutase (T-SOD), malondialdehyde (MDA), catalase (CAT), glutathione peroxidase (GSH-Px) and total antioxidant capacity (T-AOC) were measured using commercial kits (Nanjing Jiancheng Bioengineering Institute, Nanjing, China) according to the manufacturer. It is expressed as U/mg protein, and experiments were performed in triplicate.

### 2.8. Detection of Cytokines

The IPEC-J2 cells were treated with one of the four treatments previously described (CON, DQ, ST + DQ, and ST groups). After stimulation, we collected cell supernatants for the detection of cytokines. The concentrations of tumor necrosis factor-α (TNF-α), interleukin-6 (IL-6), and interleukin-8 (IL-8) were detected with enzyme-linked immunosorbent assay (ELISA) kits (Nanjing Jiancheng Bioengineering Institute, Nanjing, China) in accordance with the manufacturer’s instructions. In short, the competitive inhibition ELISA method was used to detect the content of IL-6, IL-8, and TNF-α in these four groups. Data were collected by reading optical absorption at 450 nm wavelength using a microplate reader (Tecan, Austria GmbH, Grödig, Austria).

### 2.9. Quantitative Reverse-Transcription Polymerase Chain Reaction (qRT-PCR)

IPEC-J2 cells were prepared in 12-well culture plates, cultured at a density of 5 × 10^5^ cells per well and adhered for 24 h. After four treatments (CON, DQ, ST + DQ, ST), total RNA was extracted using TRIzol total RNA isolation reagent (Invitrogen, Carlsbad, CA, USA) according to the manufacturer’s instructions. RNA quality and concentration were assessed using a NanoDrop ND-2000 Spectrophotometer (NanoDrop Technologies, Wilmington, DE, USA). Total RNA integrity was verified by electrophoresis on a 1.5% agarose gel. Approximately 1 μg total RNA per sample was used to generate cDNA by reverse transcription using the HiScript ^®^ III RT SuperMix for qPCR (+gDNA wiper) kit (Vazyme, Nanjing, China). Real-time quantitative PCR was conducted using ChamQ Universal SYBR qPCR Master Mix (Vazyme, Nanjing, China) on a CFX Connect™ Real-Time PCR Detection System (Bio-Rad, Hercules, CA, USA) with a total volume of 20 μL per reaction. All the primers used in this study are listed in Supplementary Table S1. GAPDH was applied as a housekeeping gene for normalizing gene levels. The RT-qPCR data were analyzed using the 2^−∆∆Ct^ method [23] to calculate the relative fold changes of target genes.

### 2.10. Western Blotting

The IPEC-J2 cells treated above were harvested, the total proteins were extracted, and the concentrations were detected. Loaded an equal amount of protein (30 μg) into each lane and separated them at 80 V for an initial 30 min, 120 V for 1 h. Then, the gel was transferred onto a polyvinylidene fluoride membrane (PVDF, Millipore, Merck KgaA, Darmstadt, Germany) at 330 mA for 1 h at 4 °C. After blocking with QuickBlock™ Western Blot Blocking Buffer (Beyotime, Shanghai, China) for 30 min, the blots were incubated with primary antibodies overnight at 4 °C. After washing three times with Tris-buffered saline + 0.1% Tween20 (TBST), they were incubated with secondary antibodies for 1 h at room temperature. Chemiluminescence detection was performed using a high-sensitivity ECL chemiluminescence detection kit (Vazyme, Nanjing, China) according to the manufacturer’s instructions. Immunoreactive bands were imaged using the ChemiDoc^TM^ Imaging System (Bio-Rad, Hercules, CA, USA) and quantified using ImageJ software (National Institutes of Health, Bethesda, MD, USA).

### 2.11. Statistical Analysis

All results are expressed as the mean ± standard error of the mean (SEM). Data were visualized and statistical analyses were conducted using GraphPad Prism version 9 (GraphPad Software, Inc., San Diego, CA, USA). Means were compared using one-way analysis of variance (ANOVA), followed by Duncan’s post hoc tests in SPSS (version 26.0, SPSS Inc., Chicago, IL, USA). The means of the two groups were compared using a two-tailed Student’s t-test. The 50% inhibitory concentration (IC 50) was calculated using SPSS 26.0. *p* < 0.05 was considered a significant difference.

## 3. Results

### 3.1. Stevioside Attenuated Diquat-Induced Cytotoxicity in IPEC-J2 Cells

**Figure 1:**
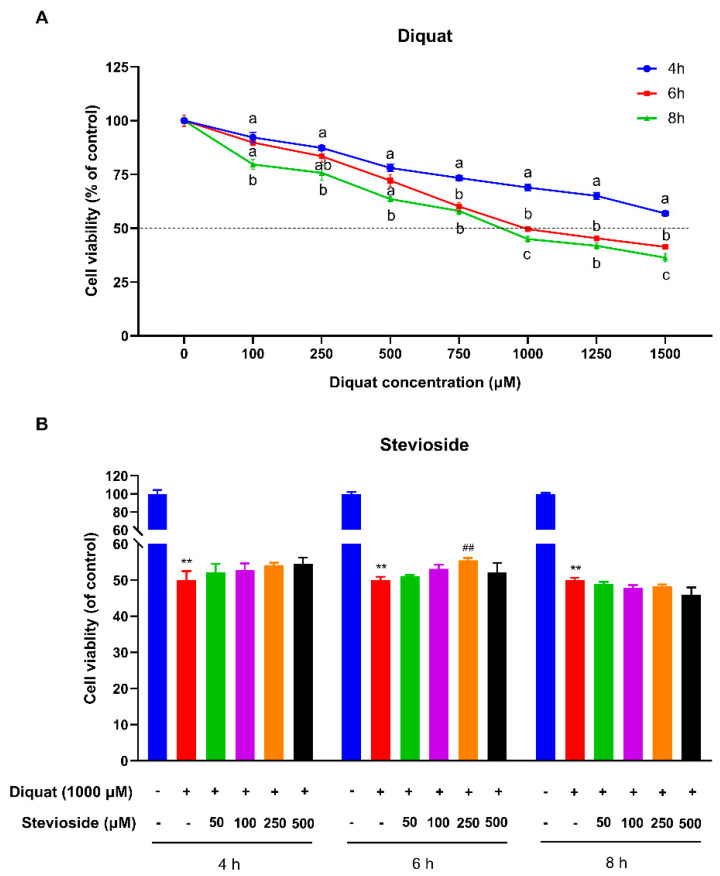
Effects of stevioside and/or diquat on the viability of IPEC-J2 cells. (**A**) IPEC-J2 cells incubated with diquat (0, 100, 250, 500, 750, 1000, 1250, or 1500 μM) for 4, 6, or 8 h. (**B**) Stevioside protects against diquat-induced cell damage of IPEC-J2 cells. IPEC-J2 cells were incubated with or without stevioside at the different concentrations (50, 100, 250, or 500 μM) for 4 h, 6 h, or 8 h, after which the medium was replaced with fresh medium containing 1000 μM diquat. After incubation for 6 h, the cell viability of IPEC-J2 cells was measured by the CCK-8 (Vazyme, Nanjing, China) assay. All data are presented as the mean ± standard error of the mean (SEM) of three independent experiments. Differences among multiple groups were compared using one-way ANOVA followed by Tukey-Kramer’s post hoc tests. Means without a common letter differ, *p* < 0.05. ** *p* < 0.01 vs. nontreated control cells; ## *p* < 0.01 vs. diquat-treated cells.

To establish a model of oxidative stress in IPEC-J2 cells, we measured the cell viability of IPEC-J2 cells after treatment with diquat by the MTT method. Compared with the control group, diquat treatment decreased cell viability in a dose- and time-dependent manner (Figure 1A). The cell viability of IPEC-J2 was significantly decreased by 50% after 6 h of treatment with 1000 μM diquat (*p* < 0.05) as compared with the control group (Figure 1A). Therefore, diquat was used to induce oxidative stress at a concentration of 1000 μM for 6 h treatment time in subsequent experiments.

As shown in Figure 1B, a 4 h or 6 h pretreatment with stevioside increased IPEC-J2 cell viability after diquat treatment, indicating that short-term stevioside pretreatment attenuates diquat-induced cell damage. Pretreatment with stevioside for 6 h more effectively protected cell viability than 4 h or 8 h pretreatment. Lower and higher concentrations of stevioside were less effective after pretreatment for 6 h, while 500 μM stevioside directly decreased IPEC-J2 cell viability and had no protective effect on diquat-induced injury. It is worth noting that diquat-attenuated cell viability was significantly reversed by the pretreatment of 250 μM stevioside for 6 h (*p* < 0.05) (Figure 1B). So, we selected a 250 μM stevioside concentration and a 6 h pretreatment time to conduct further research.

### 3.2. Stevioside Promoted Cell Proliferation in Diquat-Treated IPEC-J2 Cells

**Figure 2:**
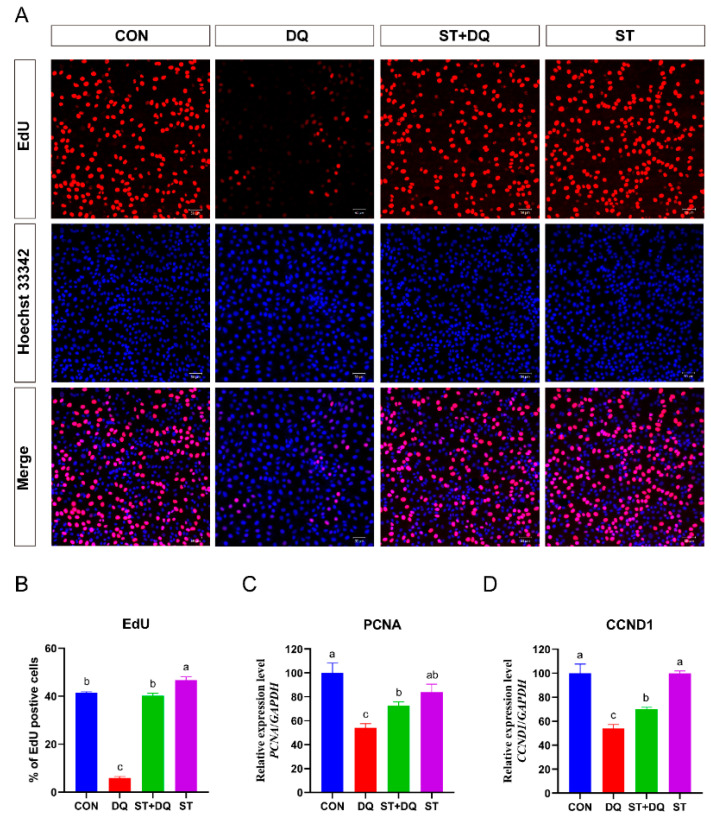
Effects of stevioside and/or diquat on the cell proliferation of IPEC-J2 cells. IPEC-J2 cells were incubated for 6 h in the presence or absence of 250 μM stevioside. Replace the medium with a fresh medium containing 1000 μM diquat and incubate for 6 h. (**A**) Cell proliferation detection was determined by the EdU assay. Proliferating cells were stained with EdU positive (red fluorescence), and cells were stained with Hoechst 33,342 (nuclear blue fluorescence). The red and blue images were merged to produce images with plink or purple fluorescence. The images were taken under a fluorescence microscope (magnification 10×, scale bar = 50 μm). (**B**) Statistical results of the proportion of EdU-positive cells. The mRNA expression of the proliferating cell nuclear antigen (*PCNA*) gene (**C**) and the Cyclin D1 (*CCND1*) gene (**D**) were measured by qRT-PCR. CON, control group, cells without being treated; DQ, diquat group, cells were only treated by diquat; ST + DQ, stevioside+diquat group, cells were pretreated with stevioside and were then treated by diquat; ST, stevioside group, cells were only treated by stevioside; EdU, 5-ethynyl-2′-deoxyuridine. Values are expressed as the mean ± standard error of the mean (SEM) of three independent experiments. Differences among multiple groups were compared using one-way ANOVA followed by Tukey-Kramer’s post hoc tests. Means without a common letter differ, *p* < 0.05.

IPEC-J2 cell proliferation was first determined using a commercially available Cell-Light EdU DNA cell proliferation kit. The result revealed that the percentages of EdU-positive cells in DQ-treated cells were significantly decreased (5.85% ± 1.39% vs. 41.51% ± 0.77%) (*p* < 0.05) compared with the control group (Figure 2A,B). In contrast, pre-treatment with stevioside significantly increased the EdU-positive cells in the ST + DQ treatment group compared with those exposed only to DQ (40.17 ± 2.20% vs. 5.85 ± 1.39%) (*p* < 0.05) (Figure 2A,B). The results of EdU incorporation experiments indicated a profound decline in fluorescence intensity upon IPEC-J2 cell exposure to diquat (1000 μM, 6h), thus signifying a noticeable reduction in DNA replication. Meanwhile, significant restoration of the DNA replication activity following diquat-induced damage was observed with the administration of stevioside at a concentration of 250 μM. To further verify the effect of stevioside on cell proliferation, the mRNA expression levels of proliferating cell nuclear antigen (*PCNA*) and Cyclin D1 (*CCND1*) were detected (Figure 2C,D). The mRNA expression levels of *PCNA* and *CCND1* decreased significantly after the 1000 μM diquat treatment (Figure 2C,D). However, the expression of *PCNA* and *CCND1* was up-regulated by stevioside pre-treatment in the IPEC-J2 cells (Figure 2C,D). These results indicated that stevioside could extremely increase the ratio of cells in the proliferation phase, dramatically promote the expression of cell proliferation-related genes, and thus promote cell proliferation.

### 3.3. Stevioside Alleviated Diquat-Induced Apoptosis in IPEC-J2 Cells

**Figure 3:**
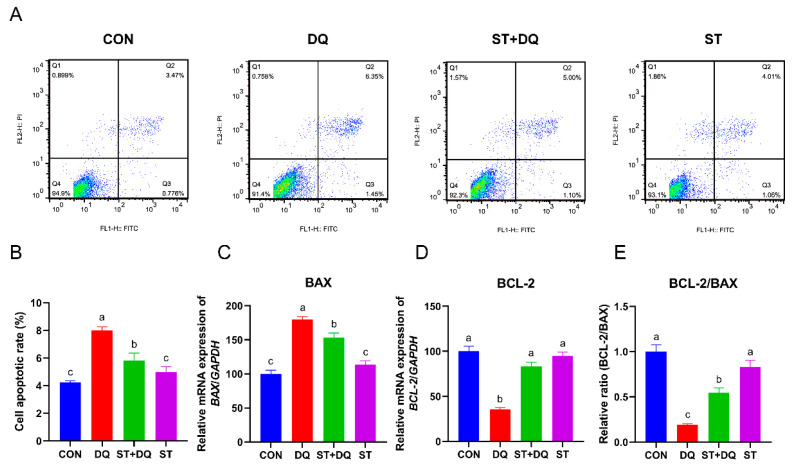
Effects of stevioside and/or diquat on the cell apoptosis of IPEC-J2 cells. IPEC-J2 cells were subjected to incubation conditions consisting of the presence or absence of 250 μM stevioside for a duration of 6 h. Subsequent to this step, the pre-existing medium was replaced with fresh medium containing 1000 μM of diquat, and the incubation was extended for an additional 6 h. (**A**) Apoptosis was determined by flow-cytometric analysis. Q1: dead cells; Q2: late apoptotic cells; Q3: early apoptotic cells; Q4: live cells. (**B**) Quantification of apoptotic cells based on flow cytometry data. Relative mRNA expression levels of BAX (**C**) and BCL-2 (**D**) were determined by qRT-PCR analysis. (**E**) Relative BCL-2/BAX mRNA expression ratio. CON, control, cells without being treated; DQ, diquat, cells were only treated by diquat; ST + DQ, stevioside+diquat, cells were pretreated with stevioside and were then treated by diquat; ST, stevioside, cells were only treated by stevioside; BCL-2, B-cell leukemia/lymphoma-2; BAX, BCL-2-associated X, apoptosis regulator; and GAPDH, glyceraldehyde-3-phosphate dehydrogenase. GAPDH was used as a normalizer. Values are expressed as the mean ± standard error of the mean (SEM) of three independent experiments. Differences among multiple groups were compared using one-way ANOVA followed by Tukey-Kramer’s post hoc tests. Means without a common letter differ, *p* < 0.05.

The IPEC-J2 cell apoptosis was measured by flow cytometry, as shown in Figure 3. Compared with the CON group, diquat incubation led to increased apoptosis, which was rescued by stevioside pretreatment (Figure 3A,B). The expression of the apoptosis-associated genes, *BCL-2*, *BAX*, and *BCL-2*/*BAX* ratio, was evaluated by qRT-PCR to further understand the antiapoptotic effect of the stevioside. qRT-PCR analysis demonstrated that diquat treatment significantly increased the mRNA abundance of *BAX* (*p* < 0.05) (Figure 3C) and reduced the abundance of *BCL-2* (*p* < 0.05) (Figure 3D) as compared with the control. Expression of the antiapoptotic factor Bax was decreased by stevioside pretreatment, whereas no obvious decrease was observed after diquat treatment alone (Figure 3C). The stevioside pretreatment dramatically increased the mRNA abundance of *BCL-2* and the ratio of *BCL-2*/*BAX* compared with the diquat treatment (*p* < 0.05) (Figure 3D,E). These results indicated that stevioside pretreatment significantly ameliorated diquat-induced apoptosis.

### 3.4. Stevioside Regulated Barrier Function in Diquat-Induced IPEC-J2 Cells

**Figure 4:**
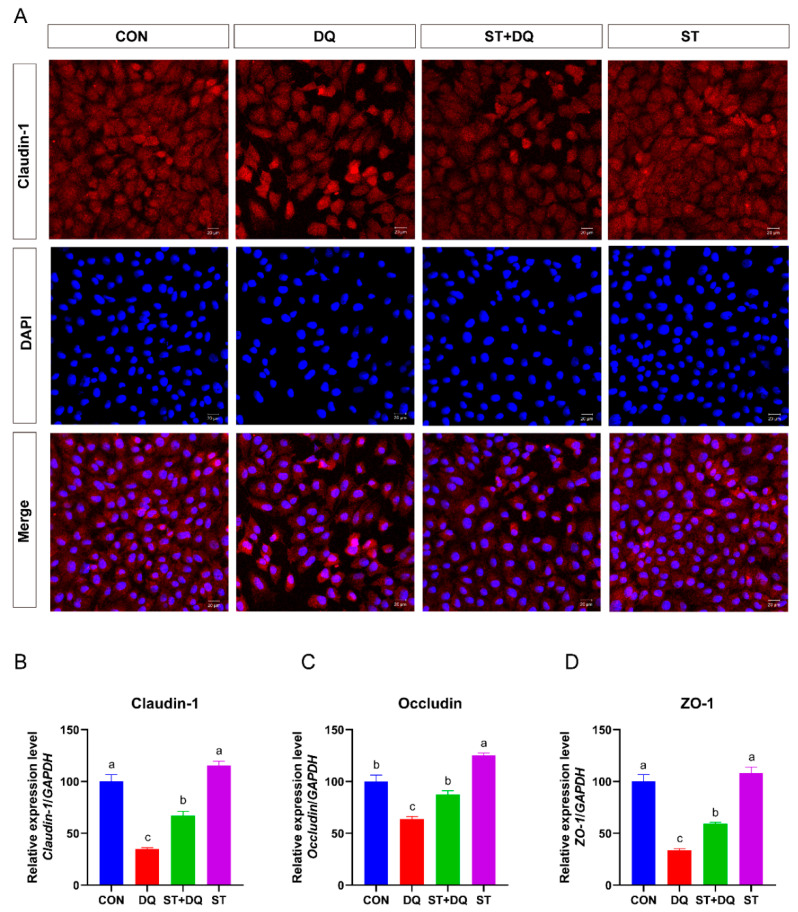
Effects of stevioside on the abundances of tight junction proteins in diquat-challenged IPEC-J2 cells. (**A**) Immunofluorescence staining localizes the tight-junction protein claudin-1. IPEC-J2 cells were seeded into 12-well plates at a density of 2.5 × 10^5^ cells/well (*n* = 3). Immunofluorescence staining of Claudin-1 (red) and DAPI (blue) in IPEC-J2 cells. The red and blue images were merged to produce images with plink or purple fluorescence. Magnification 20×, and scale bars representing 20 μm. (**B**) The mRNA expression level of claudin-1. (**C**) The mRNA expression level of occludin. (**D**) The mRNA expression level of ZO-1. CON, control, cells without being treated; DQ, diquat, cells were only treated by diquat; ST + DQ, stevioside+diquat, cells were pretreated with stevioside and were then treated by diquat; ST, stevioside, cells were only treated by stevioside. All data are presented as the mean ± standard error of the mean (SEM) of three independent experiments. Differences among multiple groups were compared using one-way ANOVA followed by Tukey-Kramer’s post hoc tests. Means not sharing a common letter are significantly different (*p* < 0.05).

To assess the role of stevioside or diquat on tight junction permeability, we determined the abundance of tight junction proteins in IPEC-J2 cells. The results showed that diquat treatment significantly decreased the abundance of tight-junction proteins (Figure 4A) and the gene expression levels of claudin-1, occludin, and ZO-1 (Figure 4B–D) as compared with the control group (*p* < 0.05). The pretreatment of 250 μM stevioside to the culture medium enhanced the gene expression abundance of claudin-1, occludin, and ZO-1 as compared with the diquat treatment (*p* < 0.05) (Figure 4B–D).

### 3.5. Stevioside Regulated the Cellular Redox State in Diquat-Induced IPEC-J2 Cells

**Figure 5:**
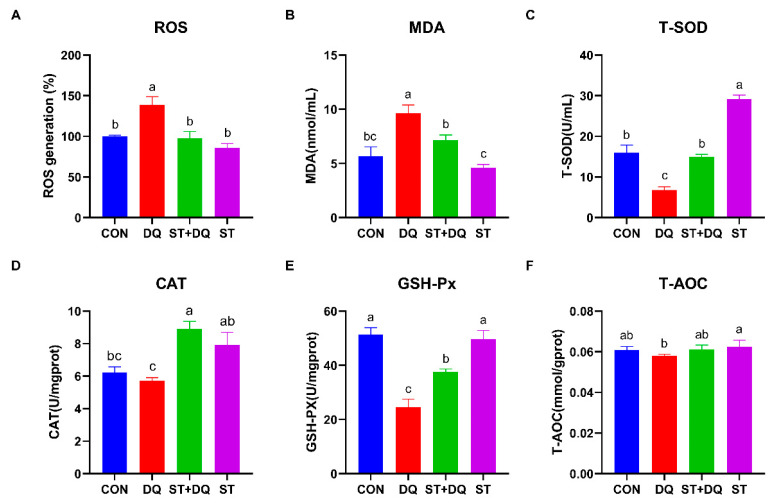
Stevioside scavenged intracellular ROS and improved the antioxidant capacity of diquat-treated IPEC-J2 cells. (**A**) The intracellular ROS content was confirmed by ELISA analysis. IPEC-J2 cells that were not subjected to DCFH2-DA incubation were utilized as blank controls. The fluorescence intensity of the cells was calculated relative to that of the control. (**B**) Cellular MDA levels; (**C**) Cellular T-SOD levels; (**D**) Cellular CAT levels; (**E**) Cellular GSH-PX levels; and (**F**) Cellular T-AOC levels. CON, control, cells without being treated; DQ, diquat, cells were only treated by diquat; ST + DQ, stevioside + diquat, cells were pretreated with stevioside and were then treated by diquat; ST, stevioside, cells were only treated by stevioside. All data are presented as the mean ± standard error of the mean (SEM) of three independent experiments. Differences among multiple groups were compared using one-way ANOVA followed by Tukey-Kramer’s post hoc tests. Means not sharing a common letter are significantly different (*p* < 0.05).

To evaluate the regulatory effect of stevioside on the redox state of diquat-induced IPEC-J2 cells, we measured intracellular ROS production capacity using a fluorescent ROS probe (DCFH-DA) (Figure 5A). The results showed that the ROS accumulation level was significantly increased after exposure to diquat (*p* < 0.05) (Figure 5A), indicating that diquat enhances the intracellular ROS burst in IPEC-J2 cells. However, pretreatment with stevioside significantly suppressed the diquat-induced ROS burst in IPEC-J2 cells.

To further investigate the redox state, the MDA was determined, which can be affected by diquat-induced ROS release. After diquat treatment alone, the level of MDA was significantly increased in IPEC-J2 cells (*p* < 0.05) (Figure 5B), indicating the occurrence of lipid peroxidation. After stevioside pretreatment, MDA content was significantly decreased (*p* < 0.05) (Figure 5B), which indicated significantly inhibited lipid peroxidation in IPEC-J2 cells. Next, we detected the levels of T-SOD, CAT, GSH-Px, and T-AOC, which serve as important indicators of the cellular redox state. After diquat treatment, intracellular T-SOD and GSH-Px levels were significantly decreased (*p* < 0.05) (Figure 5C,E), while the levels of CAT and T-AOC were decreased by 8.2%, and 4.7%, respectively, compared to the CON group (Figure 5D,F). However, pretreatment with stevioside markedly increased the T-SOD, CAT, and GSH-Px levels compared to those in the DQ group (*p* < 0.05) (Figure 5C–E), indicating the positive effect of stevioside on the redox state in IPEC-J2 cells.

### 3.6. Effects of Stevioside on Inflammatory Cytokines

**Figure 6:**
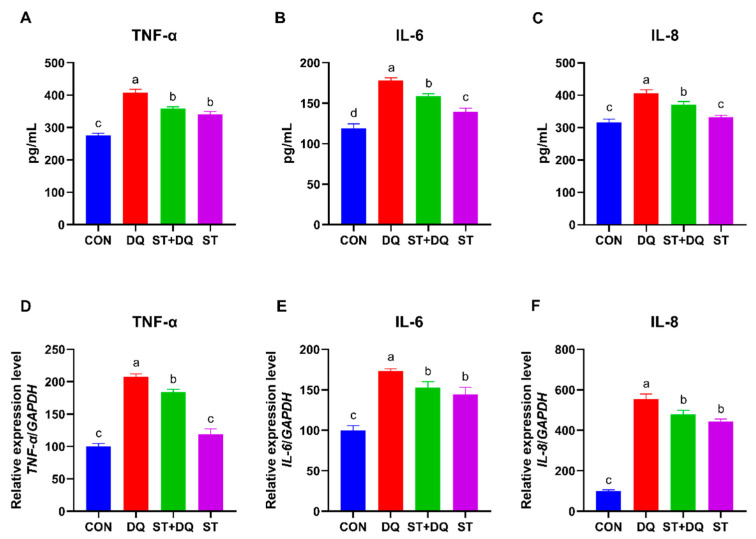
Effects of stevioside on the secretion and gene expression of inflammatory cytokines in diquat-challenged IPEC-J2 cells. The levels of TNF-α (**A**), IL-6 (**B**), and IL-8 (**C**) secretion. The gene expression levels of TNF-α (**D**), IL-6 (**E**), and IL-8 (**F**). CON, control, cells without being treated; DQ, diquat, cells were only treated by diquat; ST + DQ, stevioside+diquat, cells were pretreated with stevioside and were then treated by diquat; ST, stevioside, cells were only treated by stevioside. Values are expressed as the mean ± standard error of the mean (SEM) of three independent experiments. Differences among multiple groups were compared using one-way ANOVA followed by Tukey-Kramer’s post hoc tests. Means without a common letter differ, *p* < 0.05.

To further verify the protective effects of stevioside against intestinal inflammation, the secretion and gene expression of several inflammatory cytokines were determined in the IPEC-J2 cells. Compared with the CON group, diquat treatment caused a significant increase in the secretion of pro-inflammatory cytokines, including tumor necrosis factor-α (TNF-α), interleukin-6 (IL-6), and interleukin-8 (IL-8) (*p* < 0.05) (Figure 6A–C). In contrast to the DQ group, stevioside pretreatment dramatically decreased the secretion of pro-inflammatory cytokines, including TNF-α, IL-6, and IL-8 in the IPEC-J2 cell culture supernatant (*p* < 0.05) (Figure 6A–C). In addition, the relative mRNA abundances of *TNF-α*, *IL-6,* and *IL-8* were significantly upregulated by diquat alone treatments for 6 h in IPEC-J2 cells (*p* < 0.05) (Figure 6D,F), compared with the control group. However, they were dramatically down-regulated by stevioside pretreatment (*p* < 0.05) (Figure 6D,F). These results preliminarily inferred that stevioside could alleviate diquat cytotoxicity and decrease inflammation.

### 3.7. Stevioside Regulated NF-κB/MAPK Signaling Pathways in IPEC-J2 Cells

**Figure 7:**
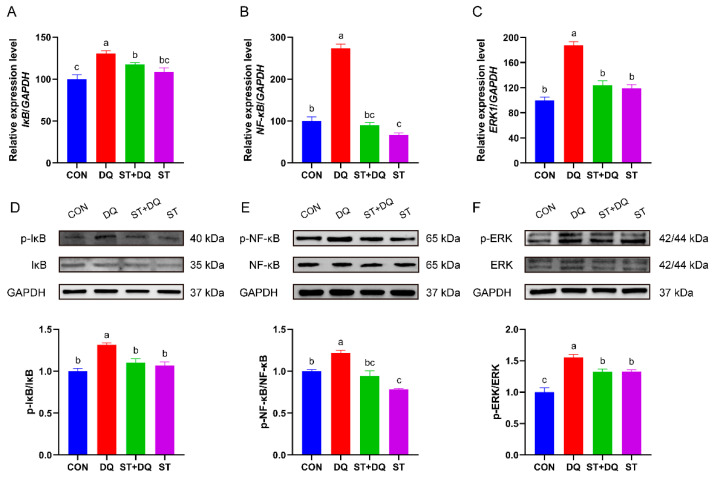
Effects of stevioside on the activation of the NF-κB/MAPK signaling pathways in diquat-challenged IPEC-J2 cells. Relative mRNA levels of (**A**) IκB, (**B**) NF-κB and (**C**) ERK1 were detected by qRT-PCR. GAPDH was used as a control. Western-blot analysis of the phosphorylation levels of (**D**) IκB and (**E**) NF-κB. (**F**) The phosphorylation level of extracellular signal-regulated kinase (ERK) 1/2. CON, control, cells without being treated; DQ, diquat, cells were only treated by diquat; ST + DQ, stevioside+diquat, cells were pretreated with stevioside and were then treated by diquat; ST, stevioside, cells were only treated by stevioside. The values presented are the means ± SEM of three independent experiments. Means without a common letter differ, *p* < 0.05.

To investigate whether the antioxidative effects of stevioside were mediated via inhibition of the nuclear factor kappa B (NF-κB)/mitogen-activated protein kinase (MAPK) signaling pathways, we evaluated the mRNA and protein abundance of NF-κB, p-NF-κB, IκB, p-IκB, ERK1/2, and p-ERK in IPEC-J2 cells. As shown in Figure 7, the diquat treatment significantly upregulated the phosphorylation levels of IκB and the mRNA abundance compared with the control and stevioside treatments in the IPEC-J2 cells (*p* < 0.05) (Figure 7A,D). In contrast, the phosphorylation levels of IκB were significantly reduced in the stevioside pretreatment group than in the diquat group (*p* < 0.05) (Figure 7A,D). In addition, the abundance level of NF-κB p65 subunit, p-NF-κB and related genes in the DQ group were significantly higher expressed compared with the CON group (*p* < 0.05) (Figure 7B,E), indicating the presence of NF-κB activation. However, stevioside pretreatment significantly reduced the mRNA and protein abundances of phosphorylated IκB and NF-κB in the diquat-challenged IPEC-J2 cells (*p* < 0.05) (Figure 7A,B,D,E). Besides, stevioside significantly decreased the mRNA and phosphorylation levels of ERK1/2 as compared with the DQ group (*p* < 0.05) (Figure 7C,F).

## 4. Discussion

In pig production, factors such as birth stress [24], weaning stress [25], feed mycotoxin pollution [26], environment [27] and social factors [28] induce the body to produce a large number of free radicals, resulting in oxidative stress. Oxidative stress has a negative impact on the production performance, health, and reproductive performance of pigs, which seriously affects the economic benefits of the pig industry [29]. Therefore, it is of great significance for animal and human health to study the prevention and treatment measures and intervention programs for porcine oxidative stress. Stevioside, a natural sweet compound from *Stevia rebaudiana* Bertoni, has been widely used as a non-nutritive sweetener in food to combat obesity and hyperglycemia [30]. Recently, stevioside has been widely reported to have antioxidant effects, which can alleviate the damage of oxidative stress on the intestinal epithelium [31]. However, whether stevioside plays an antioxidant role in IPEC-J2 cells under oxidative stress and the underlying molecular mechanism remain unclear. Therefore, this research aimed to detect the toxic effect of diquat on IPEC-J2 cell line in this study and to investigate the potential protective effect and molecular mechanism of stevioside in alleviating the oxidative stress injury of the IPEC-J2 cells induced by diquat.

Previous studies have shown that stevioside extract improves neural cell viability and proliferation [32]. Interestingly, stevioside also has the potential to be an anticancer drug because it induces a dose-dependent decline in the cell viability of breast cancer cells but promotes the proliferation of successfully surviving cells [33]. EdU is a thymidine analog that is incorporated into cells only during the S-phase of the cell cycle and thus can be used to assess cellular proliferation. Consistent with these results, DQ exposure inhibited the IPEC-J2 cells viability and proliferation according to the CCK-8 and EdU staining assays. Similarly, our results showed that stevioside pretreatment attenuated cell damage by improving cell viability and proliferation in DQ-treated IPEC-J2 cells. We also found that higher concentrations and a longer treatment time of stevioside directly decreased the viability of IPEC-J2. The present study suggests that optimal concentration and stimulation time of stevioside have the ability to enhance the viability of IPEC-J2 cells by promoting cell proliferation.

Oxidative stress is widely involved in small intestinal epithelium apoptosis [34]. Excessive oxidative stress even induces cell death [35]. A previous study determined that diquat treatment induced apoptosis and increased the level of intracellular ROS in IPEC-1 cells [36]. Similarly, the results of apoptosis detection indicated that pre-treatment with stevioside significantly decreased diquat-induced apoptosis rate and increased cell viability in IPEC-J2 cells. The present results revealed that stevioside alleviated the oxidative stress in the small intestine by inhibiting intestinal epithelial apoptosis. This indicated that stevioside is considered an effective antioxidant, which protects IPEC-J2 cells against oxidative stress-induced apoptosis.

Oxidative stress is caused by an imbalance between pro-oxidants and antioxidants [37], which often results in the disruption of the intestinal barrier integrity in the small intestinal epithelium [38]. Oxidative stress leads to impairment of the intestinal epithelial barrier, affects the digestion and absorption of nutrients, and may lead to various diseases [39]. The intestinal barrier’s integrity is crucial for maintaining intestinal homeostasis and protecting the intestinal epithelium from toxins and pathogens [40]. Tight junction proteins, including claudin-1, occludin, and ZO-1, are markers of intestinal integrity and play a crucial role in maintaining the intestinal epithelial barrier’s function [41]. Therefore, we evaluated the effects of diquat and/or stevioside on the integrity and barrier function of small intestinal epithelial cells by detecting the expression of claudin-1, occludin, and ZO-1. As expected, diquat treatment significantly decreased the expression of claudin-1, occludin, and ZO-1 compared with the CON group, while stevioside pretreatment upregulated the expression of tight junction related genes in the diquat-stimulated IPEC-J2 cells. A previous study demonstrated that stevioside improved intestinal barrier integrity, protected intestinal barrier function, and reduced inflammation in mice [42]. Consistent with these findings, our study demonstrated that stevioside prevented the increase in diquat-induced cell permeability, maintaining intestinal barrier integrity in pigs.

Reactive oxygen species (ROS) are important markers of oxidative stress, and oxidative stress can be directly assessed by measuring ROS levels [43]. A previous study demonstrated that DQ exposure increased the level of intracellular ROS and induced oxidative stress in IPEC-J2 cells [44]. Stevioside extract is recognized as an antioxidant that scavenges free radicals and exerts antioxidant activity [45]. The present results indicated that pre-treatment with stevioside significantly inhibited DQ-induced intracellular ROS accumulation, which proved that it had antioxidant properties in IPEC-J2 cells. As an indicator of lipid peroxidation [46], MDA showed a similar trend to ROS levels in this study. Diquat treatment promoted the accumulation of MDA, while stevioside pretreatment inhibited the production of MDA induced by diquat. These results indicated that the protective effect of stevioside involves antioxidant enzyme activity.

To further investigate the antioxidant mechanisms of stevioside, we evaluated the activities of some antioxidant-associated enzymes in IPEC-2 cell lysates. T-AOC normally reflects the capacity of the nonen-zymatic antioxidant defense system and is often used as a biomarker to investigate oxidative status [47]. The elevation in T-AOC after pretreatment with stevioside demonstrated that stevioside suppresses oxidative stress at least in part via the nonenzymatic antioxidant defense system. Catalase (CAT) is one of the main antioxidant enzymes, which mainly catalyzes the decomposition of hydrogen peroxide in cells to detoxify, thus playing a functional role in protecting the antioxidant system [48]. Antioxidant enzymes, such as T-SOD and CAT, can detoxify ROS into safe molecules, thus protecting cells against ROS damage [49]. In the present study, we observed that stevioside pretreatment increased the activity of T-SOD and CAT compared with diquat treatment, which also indirectly explains that stevioside could protect IPEC-2 cells against oxidative damage. GSH-Px is an important antioxidant enzyme in the cellular antioxidant defense system. The increase in its activity can eliminate the increased reactive oxidative species [50]. In the present study, we observed inhibition of GSH-Px activity by diquat, which was counteracted by stevioside pretreatment. Stevioside treatment alone increased the activity of GSH-Px to increase the antioxidant capacity. Similar results have been obtained in weaned piglets fed stevia residue extract [51]. These above results confirmed the antioxidant effect of stevioside in IPEC-2 cells.

Oxidative stress can trigger an inflammatory response, which in turn can directly induce oxidative stress [52]. The production of pro-inflammatory cytokines, such as IL-6, IL-8, and TNF-α, which are often representative hallmarks of an inflammatory response [53]. In the present study, the diquat treatment promoted the secretion of IL-6, IL-8, and TNF-α compared with the CON group and also caused a significant increase in gene expression (IL-6, IL-8, and TNF-α genes) in the IPEC-J2 cells. However, the stevioside treatment downregulated pro-inflammatory gene and protein expressions in the diquat-stimulated IPEC-J2 cells and cell supernatant. These results suggested that stevioside treatment attenuated the diquat-induced cellular inflammatory response by decreasing the production of pro-inflammatory cytokines. Consistent with our results, stevioside played an anti-inflammatory and immunomodulatory role in the human colon carcinoma cell line (Caco-2) by potentially suppressing lipopolysaccharide-induced pro-inflammatory cytokine TNF-α, IL-1β, and IL-6 productions [54]. Similarly, the latest research shows that dietary stevioside supplementation improves immunity in broilers [55]. The above results indicated that stevioside had certain anti-inflammatory and immunomodulatory effects in IPEC-J2 cells.

Pro-inflammatory cytokine production is dependent on activation of the transcription factor nuclear factor kappa B (NF-κB) signaling pathway, which regulates the expression of genes and proteins involved in the inflammatory response and immune system [56]. Therefore, we next investigated the activation of the NF-κB pathway and found that the diquat treatment significantly increased the phosphorylation of NF-κB and IκB in the IPEC-J2 cells, whereas their expression was markedly suppressed in the stevioside pretreatment group. This attenuated activation of the NF-κB pathway in turn reduced the production of pro-inflammatory cytokines, which was consistent with the changes in the cytokine levels in our results. A study has reported that the activation of the NF-κB pathway is highly correlated with the activation of the mitogen-activated protein kinase (MAPK) pathway [57]. ERK1/2 is upstream of NF-κB and the ERK1/2 MAPK pathway enhances NF-κB transcriptional activity [58]. Research suggests that stevioside attenuates LPS-stimulated inflammation by downregulating the phosphorylation levels of proteins related to the MAPK signaling pathway in mouse macrophage cell lines [59]. Similarly, our study also revealed that stevioside pretreatment significantly inhibited diquat-induced ERK1/2 phosphorylation in IPEC-J2 cells. A report demonstrated that inhibition of the MAPK signaling pathway reduced the production of pro-inflammatory cytokines [60]. Meanwhile, the NF-κB/MAPK pathway is critical for regulating inflammatory genes [61]. This means that the decrease in ERK1/2 phosphorylation in the current study may be one of the reasons for decreased IL-6, IL-8, and TNF-α expression. On the other hand, the activation of the NF-κB and MAPK signaling pathways decreased the expression of tight junction proteins in dextran sulphate sodium-induced acute colitis, which increased intestinal permeability and enhanced the inflammatory response [62]. Therefore, we accordingly speculated that stevioside improved intestinal barrier integrity and alleviated the inflammatory response by inhibiting the NF-κB and MAPK pathways in diquat-induced IPEC-J2 cells.

In this study, we confirmed the anti-oxidation and anti-inflammatory effects of stevioside in a piglet-derived cell line. This provides basic experimental data for adding stevioside to feed to improve animal growth and health. Additionally, the latest research shows that adding 400 mg/kg stevia residue extract to the diet had no significant effect on the average daily feed intake but improved the antioxidant capacity of weaned piglets and increased the relative abundance of beneficial bacteria to improve piglet health [51]. Our present study found that the pretreatment of IPEC-J2 cells with 250 μM stevioside for 6 h effectively enhanced antioxidant capacity. However, it is important to note that differences may exist between in vivo and in vitro studies. Meanwhile, adding high concentrations of stevia glycosides to feed could potentially alter the feed’s palatability and impact feed intake, highlighting the need for further research to determine the optimal dosage of stevia glycosides for use in feed. Furthermore, our findings suggest that steviosides play antioxidant and anti-inflammatory roles in cells. However, since steviosides are metabolized into steviol alcohols, stevioside metabolites are also active and have anti-inflammatory and immunomodulatory activities in colonic epithelial cells [54]. Steviosides can also directly interact with the gut microbiota [63], indicating that their mechanisms of action are multifaceted, possibly exerting antioxidant effects through multiple synergistic mechanisms. However, this study has certain limitations, including the need for validation in multiple cell lines and further in vivo studies to corroborate these findings.

## 5. Conclusions

Taken together, this is the first study to confirm that stevioside alleviated the inflammatory response, apoptosis, and exerted an antioxidant effect to alleviate diquat-induced oxidative stress through the attenuation of the activation of NF-κB/MAPK pathways. However, further research is necessary to validate these findings in vivo as well as determine the optimal percentage of stevioside to be added to pig feed.

## Data Availability

All data are presented in the article and Supplementary Materials.

## Supplementary Materials

The following supporting information can be downloaded at: https://www.mdpi.com/article/10.3390/antiox12051070/s1, Table S1: Primer sequences used for real-time quantitative PCR.
