# Supplementary material for: Stevioside Improves Antioxidant Capacity and Intestinal Barrier Function while Attenuating Inflammation and Apoptosis by Regulating the NF-κB/MAPK Pathways in Diquat-Induced Oxidative Stress of IPEC-J2 Cells
Source: Antioxidants (Basel). 2023 May 10;12(5):1070. doi: 10.3390/antiox12051070 (PMC10215602; doi:10.3390/antiox12051070)
Supplement: Supplementary file 1 [file antioxidants-12-01070-s001.zip › antioxidants-2387390-supplementary.pdf]

Table S1 Primer sequences used for real-time quantitative PCR.

Gene name	Accession number	Primer Sequence (5'-3')
PCNA	NM_001291925.1	F: AATGTTGATAAAGAGGAGGA<br>R: TAGGAGAGAGTGGAGTGGCT
CCND1	XM_021082686.1	F: CAGAAGTGCGAGGAGGAGGT<br>R: CGGATGGAGTTGTCGGTGTA
BAX	XM_003127290.5	F: CCGAAATGTTTGCTGACG<br>R: AGCCGATCTCGAAGGAAGT
BCL-2	XM_001928880.6	F: TTCTTTGAGTTCGGTGGGG<br>R: CCAGGAGAAATCAAATAGAGGC
Claudin-1	NM_001244539.1	F: AGATTTACTCCTACGCTGGT<br>R: GCACCTCATCATCTTCCAT
Occludin	NM_001163647.2	F: ATGCTTTCTCAGCCAGCGTA<br>R: AAGGTTCCATAGCCTCTCGGTC
ZO-1	XM_005659811.1	F: GAGGATGGTCACCGTGGT<br>R: GGAGGATGCTGTTGTCTCGG
TNF-α	NM_214022.1	F: CCACGCTCTTCTGCCTACTGC<br>R: GCTGTCCCTCGGCTTTGAC
IL-6	NM_001252429.1	F: ATCCAGTTGCCTTCTTGGGACTGA<br>R: TAAGCCTCCGACTTGTGAAGTGGT
IL-8	NM_213867	F: TAGGACCAGAGCCAGGAAGA<br>R: AGCAGGAAAACTGCCAAGAA
NFKBIA	NM_001005150.1	F: CCCAAGCACTCGGATACAGC<br>R: AGTCGTCATAGGGCAGCTCAT
NF-κB1	NM_001048232.1	F: CTTACACTTGGCAATCATCC<br>R: ATAGCGTTCAGACCTTCAC
ERK1	XM_021088019.1	F: CCGCTTGCCTCATTAAAGCC<br>R: TTAGACGTGGCAGCTTGGTT
GAPDH	NM_001206359.1	F: CGGAGTGAACGGATTTGGC<br>R: CACCCCATTTGATGTTGGCG
